# The Effects of Subcutaneous Rapid-Acting Insulin Aspart in the Treatment of Mild and Moderate Diabetic Ketoacidosis in Children: A Prospective Study

**DOI:** 10.7759/cureus.64241

**Published:** 2024-07-10

**Authors:** Hanieh Talebi, Zahra Razavi, Salman Khazaei

**Affiliations:** 1 Department of Pediatrics, Besat Hospital, Hamadan University of Medical Sciences, Hamadan, IRN; 2 Department of Epidemiology, Research Center for Health Sciences, Hamadan University of Medical Sciences, Hamadan, IRN

**Keywords:** rapid-acting insulin, aspart, subcutaneous insulin, intensive care, diabetic ketoacidosis

## Abstract

Background and objectives

The traditional treatment approach to diabetic ketoacidosis (DKA) involves the replacement of fluid and electrolyte deficits and a continuous intravenous infusion of regular insulin. Several clinical trials supported the administration of subcutaneous rapid-acting insulin analogs in the management of uncomplicated DKA. This study aimed to determine the effects/safety of subcutaneous rapid-acting insulin aspart injections in treating uncomplicated mild and moderate DKA in children.

Methods

In this prospective study in 2022, 25 children with mild/or moderate DKA were enrolled. The main outcome measure was median time (hours) for the resolution of ketoacidosis. Data recorded were as follows: clinical characteristics, severity of ketoacidosis and dehydration, blood glucose, sodium, potassium, creatinine, urine ketones, hospitalization’s duration, and complications. Based on the degree of dehydration, fluid deficit was replaced by sodium chloride 0.45%. Insulin aspart 0.15 units/kg subcutaneous injections were given every 2 hours in the hospital outside ICU. Blood glucose was measured hourly and blood gases every 2 hours. Ketoacidosis was considered resolved when the patient did not have nausea/vomiting, was conscious, and could eat, and blood glucose was <250 mg/dL, pH was >7.30, and/or HCO_3_ was >15 mmol/L.

Results

Of 25 DKA patients (mean age 11.06±3.89, range 4-17 years, 60% girls), 16 cases (64%) had established type 1 diabetes. Overall, 13 (52%) cases had mild ketoacidosis (average pH=7.25), and 12 (48%) cases had moderate ketoacidosis (average pH=7.15). The mean time to resolution of ketoacidosis was 11.24 hours. All but one patient met DKA recovery criteria without complications. Mild cases compared to moderate cases of DKA had a shorter duration to resolution of DKA (p = 0.04). Mean duration of hospitalization was 2.3 days. No electrolyte disturbances, hypoglycemia events, readmission or mortality, or other adverse effects were observed.

Conclusion

In children with mild and moderate DKA, subcutaneous rapid-acting insulin aspart administration was an effective, safe, and convenient treatment.

## Introduction

Diabetic ketoacidosis (DKA) characterized by the triad of hyperglycemia, ketosis, and high anion gap metabolic acidosis is a crucial medical emergency that requires proper and prompt management in a hospital setting [1]. DKA is the main serious complication of diabetes, a reason for hospitalization of a large number of people with type 1 diabetes mellitus (T1DM), and the most common cause of death in children with diabetes [2]. Although DKA commonly occurs in patients with T1DM, patients with type 2 diabetes are susceptible to this condition in stressful situations such as trauma, surgery, or infection [3]. Effective restoration of circulating fluid volume and intravenous (IV) insulin infusion are the cornerstones of DKA management. Correction of electrolyte disturbances, identification and treatment of predisposing conditions, careful clinical evaluation, and monitoring of vital signs and biochemical parameters are critical [4]. Continuous IV infusion of low-dose regular insulin in an intensive care unit (ICU) or a high-dependency unit where appropriate intensive nursing care and access to laboratory test results are available is the standard protocol of insulin therapy in patients with DKA [5,6]. Compared to subcutaneous injections, infusion of IV insulin and ICU admission require more drugs and equipment and more care provided by experienced nursing staff, which create a significant economic burden. IV insulin therapy is also technically more difficult with higher rate of complications of venous treatment, including thrombophlebitis [7,8]. Furthermore, ICU admission may cause emotional distress to patients and families, as well as increase the risk of long-term mortality [7].

Considering the aforementioned disadvantages, there is a growing interest in DKA treatment with subcutaneous insulin injection as an alternative to IV insulin infusion. Data on prospective randomized studies have demonstrated that the use of subcutaneous insulin injection is a valid and effective alternative to IV insulin without a significant difference in the outcome in uncomplicated mild and moderate DKA [2,8-15].

This prospective study was designed with the aim of strengthening the current clinical evidence regarding the efficacy and safety of rapid-acting subcutaneous insulin aspart injection in pediatric patients with mild and moderate DKA.

This article was previously posted to the Research Square preprint server on March 19, 2024 (https://doi.org/10.21203/rs.3.rs-4103362/v1).

## Materials and methods

Study design

This prospective study was conducted at Besat Hospital in Hamadan, Iran, in a large tertiary referral and teaching center where all adolescents and children with DKA are admitted. As per our hospital’s routine, children with DKA are treated with IV insulin in the ICU setting. Rather, in this study conducted in 2022 over a one-year period, patients were treated with the administration of insulin aspart subcutaneously while staying inside the hospital but outside the ICU.

Ethical consideration

The Research Ethics Committee of Hamadan University of Medical Sciences approved the study (IRB No: IR.UMSHA.REC.1401.318). Written informed consent was obtained from the parents or legal guardians of the patients in the study.

Study criteria

Pediatric patients with mild and moderate DKA (newly diagnosed or known case of T1DM) were enrolled. The primary outcome was the duration of insulin therapy (hour) to resolution of DKA.

Procedure

At the time of hospitalization, a complete medical history was taken, and a physical examination including heart rate and breathing rate, level of consciousness, and the degree of dehydration (evaluated based on clinical examination) was performed. Samples were taken for blood glucose, venous blood gas, complete blood count, serum biochemistry profile, and urine test for ketones. Patients with mild and moderate DKA (pH of 7.2-7.3 and pH of 7.1 to <7.2, respectively) were included in the study and were admitted to our pediatric department outside the ICU. The detailed laboratory results and the volume of administered fluids, insulin, and electrolytes were recorded in a DKA flowchart. Based on the degree of dehydration, ranging from 6.5% to 8.5%, fluid deficit was replaced by sodium chloride 0.45% (1/2 NS, also known as half-strength normal saline) [16], and in the absence of dehydration, oral fluid therapy was started. Treatment with insulin aspart 0.15 units/kg subcutaneously was administered at least 1 hour after fluid resuscitation and then continued every 2 hours. Vital signs and neurological status were monitored regularly. Blood glucose was measured every 1 hour and blood gases every 2 hours. Subcutaneous insulin injection every 2 hours was discontinued once DKA was resolved. Those who did not respond to the treatment were transferred to the ICU to receive standard treatment of IV regular insulin.

The laboratory criteria for the presence and severity of DKA were determined based on the International Society for Pediatric and Adolescent Diabetes (ISPAD) Clinical Practice Consensus Guidelines 2018 [4,17]. DKA diagnosis was established when blood glucose was ≥250 mg/dL with a venous pH of <7.3 and/or a bicarbonate (HCO_3_) level of <15 mmol/L, and ketonuria was present. For the purpose of this study, the resolution of DKA was considered when venous pH was above 7.3 and/or serum bicarbonate was greater than 15 mmol/L, the patient was fully conscious, was able to eat, and had no nausea and vomiting [18].

Then the newly diagnosed patients were placed on the multiple daily insulin injections regimen. Those with established T1DM were placed on their previous treatment regimen. Data regarding clinical characteristics, severity of dehydration, severity of ketoacidosis, blood glucose, sodium, potassium, blood urea nitrogen (BUN), creatinine, urine ketones, length of hospitalization, and complications were analyzed.

Inclusion Criteria

All pediatric patients with established T1DM or new cases that met all the criteria for DKA were included in the study.

Exclusion Criteria

Patients with abnormal sensorium, those with other underlying medical conditions, those taking medications other than insulin or requiring other interventions, those who had evidence of an infectious disease, and those who received insulin or fluids prior to admission to our center were excluded. The severity of DKA was categorized by the degree of acidosis: Mild (pH of 7.2-7.3), moderate (pH of 7.1 to <7.2), and severe (pH < 7.1) [18].

Sample size calculation

For this proof-of-concept research, we assumed a larger effect size of around 1.5 units, a low variability with a standard deviation of around 2 units, significance level of 0.05, and power of 80%, which gave us the sample size of 25.

Statistical analysis

Statistical analysis was performed using SPSS Version 23 (IBM Corp., Armonk, NY). Quantitative characteristics were presented as means and standard deviations (SD). Qualitative data were expressed as number and percent. Paired t-test was used to compare quantitative variables, and McNemar's test was used to compare qualitative variables before and after the intervention. A p-value of <0.05 was considered statistically significant.

## Results

A total of 25 patients were included, of whom 15 (60%) were female. The overall mean age (and SD) was 11.6 ±3.89 (range 4-17) years. There was no significant difference between the mean age of boys and girls (p=0.23). Of patients, 13 (52%) cases were in the mild ketoacidosis group, and 12 (48%) cases were in the moderate ketoacidosis group. The majority (16 or 64%) of DKA episodes occurred in patients known to have T1DM. Tables 1, 2 present the basic qualitative and quantitative characteristics of the patients in the study.

**Table 1:** Basic qualitative characteristics of the patients in the study (n=25)

Variable		Number (Percent)
Sex	Female	15 (60%)
	Male	10 (40%)
DKA	Mild	13 (52%)
	Moderate	12 (48%)
History of diabetes	Known	16 (64%)
	New	9 (36%)

**Table 2:** Basic quantitative characteristics of the study patients (n=25) *P-values < 0.05 were considered significant DKA, diabetic ketoacidosis; SD, standard deviation

Variable		Mean ±SD	P-value
Age (years)	Female	11.3 ±4.43	0.23
	Male	9.4 ±3.42	
Duration of hospitalization (days)	Mild DKA	2.08 ±0.64	0.12
	Moderate DKA	2.58 ±0.9	
Duration of ketoacidosis or time to resolution of DKA (hours)	Mild DKA	9 ±6.42	0.04*
	Moderate DKA	13.67 ±4.5	

The mean blood glucose before treatment in the mild group was 439 mg/dL and that in the moderate group was 474.15 mg/dL. There was no significant difference between mean blood glucose levels based on the severity of DKA before the intervention (p=0.52). The mean pH was 7.25 in the mild group and 7.15 in the moderate group. The mean HCO_3_ was 12.04 mmol/L in the mild group and 9.28 mmol/L in the moderate group. The biochemical and laboratory characteristics of the two groups with mild and moderate ketoacidosis and the comparison of the two groups are shown in Tables 3, 4.

**Table 3:** Comparison of the biochemical and laboratory characteristics of children with mild and moderate DKA before and after the intervention (n=25) ^α^The intervention was conducted over an average of 9 hours in the mild group and 13.67 hours in the moderate group. *P-values < 0.05 were considered significant DKA, diabetic ketoacidosis; BUN, blood urea nitrogen; SD standard deviation

Variable	Category	Before the Intervention		After the Intervention^α^	
		Mean ± SD	P-value	Mean ± SD	P-value
pH	Mild DKA	7.25 ± 0.44	<0.001*	7.37 ± 0.05	0.48
	Moderate DKA	7.15 ± 0.023		7.36 ± 0.04	
Bicarbonate (mmol/L)	Mild DKA	12.04 ± 4.72	0.04*	15.57 ± 4.17	0.38
	Moderate DKA	9.28 ± 9.04		14.35 ± 2.4	
Blood glucose (mg/dL)	Mild DKA	474.15 ± 164.42	0.52	272.15 ± 70.58	0.62
	Moderate DKA	439 ± 91.94		288.08 ± 84.3	
Sodium (mEq/L)	Mild DKA	139.8 ± 2.69	0.23	139.15 ± 3.48	0.51
	Moderate DKA	138.13 ± 2.34		138.33 ± 2.61	
Potassium (mEq/L)	Mild DKA	3.87 ± 0.53	0.13	3.75 ± 0.44	0.22
	Moderate DKA	4.34 ± 0.95		3.95 ± 0.4	
BUN (mg/dL)	Mild DKA	13.92 ±6.02	0.24	15.08 ± 5.56	0.30
	Moderate DKA	16.67 ± 5.33		17.17 ± 4.06	
Creatinine (mg/dL)	Mild DKA	1.04 ± 0.28	0.89	0.92 ± 0.21	0.74
	Moderate DKA	1.03 ± 0.22		0.9 ± 0.11	

**Table 4:** The biochemical and laboratory characteristics of the patients before and after the intervention (n=25) ^α^The intervention was conducted over an average of 9 hours in the mild group and 13.67 hours in the moderate group. *P-values < 0.05 were considered significant BUN, blood urea nitrogen

Variable	Before Intervention	After the Intervention^α^	P-value
Blood glucose (mg/dL)	457.2 ± 133	279.8 ± 76.2	<0.001*
Sodium (mEq/L)	138.78 ± 2.5	138.76 ± 3.05	0.97
Potassium (mEq/L)	4.09 ± 0.78	3.84 ± 0.42	0.08
pH	7.20 ± 0.63	7.36 ± 0.44	<0.001*
HCO_3_ (mmol/L)	7.72 ± 3.98	14.98 ± 3.4	<0.001*
BUN (mg/dL)	15.24 ± 5.7	16.08 ± 4.9	0.28
Creatinine (mg/dL)	1.03 ±0.246	0.91 ±0.166	<0.001*

The mean duration of treatment with subcutaneous insulin aspart until improvement of ketoacidosis was 11.24 hours. Mild DKA was associated with faster resolution of acidosis (9 versus 13.67 hours, p = 0.04). All but one patient met DKA recovery criteria without any complications. The mean duration of hospitalization was 2.3 days. There was no significant difference between the duration of hospitalization based on the severity of DKA (p = 0.12). There were no electrolyte disturbances, hypoglycemia events, mortality, or other complications with subcutaneous insulin aspart administration. One patient was admitted more than once due to discontinuation of insulin administration. Patient satisfaction with this treatment protocol in the group with mild ketoacidosis was 76.9% and that in the group with moderate ketoacidosis was 91.6%, especially because they were not hospitalized in the ICU.

## Discussion

In this study, we present data on the use of subcutaneous rapid-acting insulin aspart in the treatment of children and adolescents with mild and moderate DKA in a large referral public hospital. Our results indicated that subcutaneous injection of insulin aspart every 2 hours, which removed the need for ICU admission, was found to be an effective, safe, and convenient alternative for the treatment of mild-to-moderate DKA in pediatric patients. This finding confirms the results of previous studies regarding the safety, cost-effectiveness, and feasibility of subcutaneous insulin administration, which similarly removed the need for ICU admission without causing any mortality or serious adverse effects in the management of patients with mild-to-moderate DKA. The results of previous studies suggest that subcutaneous insulin also reduces the discomfort of patients [7,14,15,19]. In this regard, Ersöz et al. demonstrated that the treatment of mild and moderate DKA with subcutaneous insulin lispro is equally effective and safe compared with regular IV insulin [20]. Similarly, Vincent and Nobécourt stated that treatment of mild and moderate DKA with subcutaneous insulin lispro is a practical alternative protocol to conventional IV infusion of regular insulin [19]. Andrade-Castellanos et al. reviewed and compared the results of five randomized controlled trials conducted primarily in adults on the effects of subcutaneous insulin analogs versus regular IV insulin for the treatment of DKA. They found that there were no significant advantages or disadvantages between the two treatment protocols [21].

During the COVID-19 pandemic, critical care resources were limited and there was a fear of the risk of transmission of COVID-19 infection to the hospitalized young people with T1DM or type 2 diabetes. Based on considerable evidence on the safety and efficacy of subcutaneous insulin therapy in the treatment of mild and moderate ketoacidosis [7,14,15,20,22], ISPAD in a scientific guideline recommended that in some critical situations, such as the COVID-19 pandemic, subcutaneous insulin therapy is a valid alternative to IV insulin in patients with non-severe DKA. This guideline notes that the mentioned strategy allows these patients to be admitted to medical wards far away from the patients with COVID-19 to reduce the risk of transmission of infection and to provide more ICU beds for patients with COVID-19 [13].

We observed that the time taken to the resolution of ketoacidosis and clinical improvement (11.24 ±5.8 hours) was similar to a previously published report from our center [22]. As expected, the duration of treatment was longer in the moderate group compared to the mild group (13.67 hours versus 9 hours). Similar to the results from this current work, in a study by Bali et al., DKA in the group treated with subcutaneous insulin was resolved 2.83 hours earlier than in the IV group [23].

In agreement with recent reports by Andrade-Castellanos et al. [21], our alternative treatment method was also accompanied by another benefit, which was a short hospital course (mean 2.3 days). In a previous study in our center, we found that the length of hospitalization in patients treated with subcutaneous insulin was less than those treated with IV insulin (3.38 days versus 4.42 days) [22]. However, some previous authors did not find a significant change in the length of hospitalization in patients treated with subcutaneous insulin [2,4,11,13]. In the study conducted by Umpierrez et al., no difference was observed in the duration of hospitalization, but the cost of admission was lower in the group treated with subcutaneous insulin [6]. The explanation for why the length of stay of our patients in the hospital was less than similar studies may be that 64% of the patients in this research were patients known to have T1DM and thus were discharged earlier than the patients who were new cases of T1DM who needed to receive basic training for living with diabetes.

Consistent with the results from previous studies [2,20,22], in the present work, none of the patients exhibited biochemical and clinical signs suggestive of hypoglycemia, hypokalemia, and hyponatremia, which is one of the important advantages of this alternative method [18,20]. By using IV insulin, significant hypoglycemia and hypokalemia continue to be areas of concern [24].

There were no cases of acute kidney failure or renal tubular necrosis, which are one of the complications of DKA [25]. No other adverse effects were found in association with subcutaneous insulin aspart. One case with moderate DKA was non-responsive during the subcutaneous treatment with insulin aspart and was transferred to the pediatric ICU (PICU) and treated with IV insulin protocol.

Another notable observation was that the majority of patients and parents felt satisfied that they were not admitted to the PICU. PICU admission exposes children and their families to a wide range of stressful experiences with long-term psychological consequences, cognitive impairment, and emotional and behavioral problems because of what happens to the patients themselves and also the events that they witness for other patients in the special care department [26-30]. Subcutaneous insulin therapy, which does not require ICU admission, may effectively minimize or prevent the long-term psychological effects of ICU admission.

Finally, avoiding ICU admission of patients with uncomplicated DKA would make more ICU beds available for patients with more complex conditions, reducing healthcare costs and improving resource utilization.

Study limitations

Despite all the advantages mentioned previously, it can be argued that the current protocol was performed on carefully selected patients under the supervision of trained personnel and an experienced medical team familiar with the treatment of children and adolescents with DKA in the endocrinology department where rapid return of laboratory test results was possible. The implementation of this treatment method in medical centers without favorable facilities may lead to the deterioration of the clinical condition of patients. This creates the first limitation of this study, and additional prospective studies are required to assess the generalizability, benefits, and disadvantages of subcutaneous versus standard protocol of slow IV insulin infusion therapy in the management of DKA. Secondly, it was not possible to measure blood ketone in our center. Considering that the level of serum ketone was not a criterion for improvement of ketoacidosis [5], it does not seem to have created a problem in our results. Thirdly, the sample size was small. Fourthly, repeated injections of insulin every 2 hours can be painful and inconvenient for a child. Nevertheless, our study’s results corroborate and strengthen the knowledge of treating patients with mild and moderate ketoacidosis with subcutaneous insulin.

## Conclusions

Our results showed that the implementation of the protocol for subcutaneous injections of rapid-acting insulin aspart encourages an acceptable recovery time for mild or moderate ketoacidosis and leads to timely discharge of pediatric patients without causing any adverse effects. In addition, since IV insulin treatment is technically more difficult and associated with a higher rate of side effects, this easy alternative method, which is implemented outside an ICU setting, may be a more appropriate treatment strategy. This practice also increases the availability of ICU beds for other critically ill patients. Our study supports the alternative practice of simple, safe, and convenient subcutaneous insulin therapy in the treatment of children with mild-to-moderate ketoacidosis.
